# Comparing the productive vocabularies of grey parrots (*Psittacus erithacus*) and young children

**DOI:** 10.1007/s10071-024-01883-5

**Published:** 2024-06-24

**Authors:** Tereza Roubalová, Lucie Jarůšková, Kateřina Chládková, Jitka Lindová

**Affiliations:** 1 Faculty of Humanities, Charles University, Pátkova, 2137/5, 182 00 Prague 8, Czech Republic; 2 Faculty of Arts, Charles University, nám. Jana Palacha 1–2, 116 38 Prague 1, Czech Republic; 3 Institute of Psychology, Czech Academy of Sciences, Voršilská 1, 110 00 Prague 1, Czech Republic

**Keywords:** Communicative development, Grey parrot, Children, Vocabulary, Language, Cross-species comparison

## Abstract

Due to their outstanding ability of vocal imitation, parrots are often kept as pets. Research has shown that they do not just repeat human words. They can use words purposefully to label objects, persons, and animals, and they can even use conversational phrases in appropriate contexts. So far, the structure of pet parrots’ vocabularies and the difference between them and human vocabulary acquisition has been studied only in one individual. This study quantitatively analyses parrot and child vocabularies in a larger sample using a vocabulary coding method suitable for assessing the vocabulary structure in both species. We have explored the composition of word-like sounds produced by 21 grey parrots (*Psittacus erithacus*) kept as pets in Czech- or Slovak-speaking homes, and compared it to the composition of early productive vocabularies of 21 children acquiring Czech (aged 8–18 months), who were matched to the parrots by vocabulary size. The results show that the ‘vocabularies’ of talking grey parrots and children differ: children use significantly more object labels, activity and situation labels, and emotional expressions, while parrots produce significantly more conversational expressions, greetings, and multiword utterances in general. These differences could reflect a strong link between learning spoken words and understanding the underlying concepts, an ability seemingly unique to human children (and absent in parrots), but also different communicative goals of the two species.

## Introduction

Parrots have an astonishing ability to imitate human speech, yet ‘parroting’ is commonly understood to mean just mindless repetition. Research on interspecies communication shows that parrots can use some words meaningfully and, to a limited extent, even apply some simple vocal syntactic rules (e.g. Colbert-White et al. 2016; Pepperberg 2007). Although the word-like repertoire of a couple of exceptionally linguistically endowed grey parrots has been mapped in substantial detail, little is known about what kinds of words grey parrots tend to pick up and produce, or how that differs from what human children spontaneously learn (Colbert-White et al. 2011, 2016; Kaufman et al. 2013; Pepperberg 2002, 2007). A systematic investigation of the speech repertoire of a larger sample of home-raised parrots that have not been deliberately trained to speak and subsequent comparison with children’s linguistic behaviour could help us better understand not only parrot’s language-like skills but also the preferences, biases, and tendencies they exhibit in relation to communication. A better understanding of how grey parrots absorb words from available linguistic input could, in turn, tell us something about the functions which an acquired system of communication might have in nonhuman animals. Moreover, a general, first overview of the speech repertoire could inspire further hypothesis-driven research of the language-like learning mechanisms of grey parrots. This study therefore explores the differences and similarities in the composition of productive vocabulary of spoken language of infants and toddlers (aged 8–18 months) living in Czech-speaking households and the productions of word-like sounds of grey parrots *(Psittacus erithacus)* kept as pets in Czech- and Slovak-speaking households.

Spoken language is a system of communication in which speech sounds, syllables, and words are combined in a structured way based on phonological and morphosyntactic relations (Höhle and Weissenborn 1999). The number and type of words language users can employ in production comprise their productive vocabulary (Teichroew 1982). Together with the receptive vocabulary (which describes the understanding of language), the size of productive vocabulary is one of the most widespread and relatively straightforwardly assessed measures used to describe communicative development in humans. It works relatively well despite possible biases due to parental reporting choices in home settings or methodological limitations of laboratory settings (e.g., Fenson et al. 2007). While they do not always meet the requirements for a linguistic use of these sounds, grey parrots kept in a linguistic environment often produce word-like sounds resembling human spoken words. In this exploratory study, which is a pilot attempt to investigate the differences and similarities in communicative development of different species, we compared the productive vocabulary of very young children with the production of word-like sounds in pet grey parrots using a methodological approach that is suitable for both species and allows for a direct comparison. Because we wanted to compare the structure of speech production of the two species, we matched parrots to children by the number of ‘words’ or multiword utterances they produced, as reported by their caregivers and parents, respectively. Size-matching was also why we chose this particular age range in children.

Although we were comparing two phylogenetically distant species that naturally have different life histories, we believe that pet parrots and children are exposed to a similar language input. Children acquire the ambient language through exposure. In other words, in the course of childhood, children learn to understand and speak the language that is spoken around them and towards them, in their family, by parents, caregivers, or peers. Home-raised parrots usually spend most of their lives in a human social group, so their human family is the only social group they have ever known. It is thus likely that they view humans as their partners or flock members (Tygesen and Forkman 2023), while caregivers consider them part of their family (Anderson 2014). Although parrots are housed in cages, they are usually for part of the time released and allowed to move around the household and explore (Anderson 2014), and while small children travel outside their home, they usually stay with their relatives who are thus still the main source of their language input.^1^ The linguistic environment of both children and pet grey parrots thus consists mainly of household communication with and among their caregivers and other family members. On top of that, human adults use affective and relatively simple language when speaking to both children and pet parrots (children: Soderstrom 2007, parrots: Xu et al. 2013).

Despite this relative similarity in the type of linguistic environment encountered by children and parrots, there may still exist important differences in the amount of linguistic and social activity directed at them, the amount of contact with speakers outside the household, and in their general cognitive abilities the two species can use to acquire language. From the sociocognitive and pragmatic perspective, the ontogeny of language in children reflects possibly unique human cooperative communicative intentions, which in some cultural environments include focusing on and naming objects jointly with an adult. In this respect, human language might, since early stages, differ from analogous communication systems in other animals. This may be evident from the early stages of children's language use, when the level at which children use language does not yet exceed the complexity of some communication systems in other species (see, e.g., comparative studies with chimpanzees: Bard et al. 2021; Greenfield and Savage-Rumbaugh 1993).

## Vocabulary composition: the first words in language acquisition

There is an abundance of crosslinguistic studies on children’s early vocabulary: researchers have examined the size of vocabularies which children understand and produce, their growth rate, composition, and the development of relations between word forms and meanings (e.g., Clark 1993; O’Grady 2005; Tomasello 2003). It has been shown that by about six months of age, children will have formed the first word–meaning mappings and several months later, they start producing the first words with referential meaning (Bergelson and Swingley 2012; Majorano and D’Odorico 2011). In fact, children sometimes begin to communicate referentially about objects and events using gestures even before they can speak, for example by pointing at various food items they want (Tomasello 1999).

Several studies have shown that both deliberately trained and untrained captive parrots can communicate referentially (trained: Giret et al. 2010; Pepperberg 2002; untrained: Colbert-White et al. 2016) or at least use labels in appropriate contexts (e.g., untrained: Colbert-White et al. 2011; trained: Pepperberg 2007).

In terms of the composition of early vocabularies, children during the first two years of life tend to use labels for persons and things which are part of their everyday lives; such labels make up over one-half of their early vocabulary (e.g., Kern 2007; O’Grady 2005; Rescorla 1981). In terms of content, children’s early vocabularies are rather variable, but according to studies on English-, Czech-, and Slovak-speaking children, the vocabularies tend to consist of labels for persons, animals, food, and various kinds of objects such as body parts, vehicles, toys, clothing, and household items (English: O’Grady 2005; Rescorla 1981; Czech: Votavová and Smolík 2010; Slovak: Kesselova 2008). Studies on other languages show a similar bias towards labelling persons, animals, and objects in early language development (Bornstein et al. 2004; Gentner 1982; Kern 2007), where object labels outnumber labels for activities, states, or social phrases. Activity labels are related to children’s daily routines, games, or successes and failures (e.g., *pee*, *peek-a-boo, did it, uh-oh*). They make up around 20% of children’s early vocabulary (Clark 1993). Furthermore, young children also use conversational formulas, such as greetings or placement expressions (e.g., *bye-bye, up, there*) (Kesselova 2008; O’Grady 2005). Such expressions can usually be found among the first 10 to 20 words, but they tend to be restricted to a particular context, such as *bye* produced only when someone leaves (Clark 1993). Regarding the form of the first words, it has been noted that they often resemble real sounds from the outside world, such as animal vocalisations, mechanical noises, or sounds from nature (e.g., *sss* for snakes, *choo, choo, choo* for trains) (Kesselova 2008; Laing et al. 2017; Menn and Vihman 2011).

It is important to note that the semantic categories of children’s first words seem to be less clearly defined than in adults, indicating that words in the early vocabulary have changing or uncertain meaning boundaries. Until 24 months of age, children often overextend the meaning of words, that is, they tend to apply an expression to a wider group of persons and/or objects than an adult would when using the same linguistic item (Clark 1993; Rescorla 1980). For instance, six-month-old infants who acquire the English expression ‘mama’ tend to use it to refer to family members including parents, grandparents, siblings, aunts, and even dogs in a so-called ‘wanting context’ (Goldman 2001).

The communicative and cognitive abilities of grey parrots have been studied notably by Irene Pepperberg (e.g., Pepperberg 2002, 2007), who showed that some language-trained birds are capable of labelling up to a hundred objects, seven colours, five shapes, and four materials. Parrots can refer to object properties and abstract concepts, make requests, and faithfully imitate animal vocalisations and environmental sounds (Colbert-White et al. 2011; Giret et al. 2009; Pepperberg 1990, 2002, 2007). Parrot’s spontaneous vocabulary has been systematically described in detail for the individual Cosmo (Colbert-White et al. 2011), whose caregiver spoke to her and labelled objects similarly to how an adult would label objects to a child. Cosmo used mainly conversational phrases (‘How are you?’, ‘Goodbye’, etc.), names of persons and animals (including herself), and she could make various requests (Colbert-White et al. 2011).

## Communicative functions of the first words

The predominance of objects among children’s first words might stem from the importance of certain objects in their lives and cultural environment. From early on, children play with objects and learn about them, be it toys or ordinary household objects (Chase 1992). This focus translates into a certain sequence of acquisition of certain language elements. Even before children start speaking, new words attract their attention to objects. They help them discover various similarities and differences between objects based on their size, shape, and other characteristics, such as colours (Fulkerson and Waxman 2007; Waxman and Leddon 2011). Moreover, object labels are considered to be suitable starting points when learning a referential system, such as the human language, because they refer to individual concrete referents (Gentner and Boroditsky 2001). The learning of object labels typically takes the form of triadic communication (adult–child–object) where joint attention, which develops at about nine months of age (Striano and Rochat 1999; Tomasello 1999), plays a pivotal role. It has been proposed that triadic communication might be a characteristic that distinguishes human communication from the communication systems of other species. It requires cooperation between interaction participants, including mutual attention, and perceived need to share intentions, information, or engage in shared activity (Carpenter 2011; Tomasello 1999).

It has been reported that object play is common and intrinsically rewarding not only in humans but also in hand-reared grey parrots (Auersperg et al. 2014). It is an activity that helps them practise their motor skills and motivates innovative problem solving (O’Hara and Auersperg 2017). O’Hara and Auersperg (2017) conclude that object play in parrots persists into adulthood: it can help them learn necessary life skills and retain their flexibility in changing environments. There is, however, no clear evidence showing that parrots are interested in learning labels in terms of referential signals when such labelling is not linked to a direct reward (Bradbury and Balsby 2016). Pepperberg and McLaughlin (1996) suggest that in methodical language training, grey parrots must use joint attention to successfully obtain object labels and comprehend them, but we have very little information about the role of joint attention in acquiring new word-like sounds by pet parrots.

Based on the above findings, we formulate our first prediction:

Prediction 1: Children’s productive vocabularies contain more object labels than parrots’ speech repertoires.

Still, vocal learning in grey parrots has other, well-documented functions. In the wild, parrots use duets and contact calls, which are sometimes associated with individual identity and compared to names (Berg et al. 2012; Wanker et al. 2005), in order to strengthen the bond with their mates and to maintain contact (Bradbury 2003; Dahlin and Wright 2012; Todt and Naguib 2000). Research has shown that parrots gradually start to imitate the vocalisations of their partners (e.g., Hile et al. 2000; Scarl and Bradbury 2009). A similar phenomenon can be observed in groups, where newly introduced individuals adopt the vocalisations of other group members (e.g., Hile and Striedter 2000). In that context, Giret et al. (2010) pointed out that the relationship between a parrot and its caregiver can play an important role in a parrot’s willingness to learn words and communicate. It is thus possible that talking parrots use speech to establish a closer relationship with their human caregivers as an alternative to a pair bond such as they would form in the wild (Colbert-White et al. 2011; Langford 2017). Parrot’s efforts to attract the attention of others and their learning how to initiate communication may be thus motivated by relationship maintenance. Moreover, the tendency to imitate vocalisations of other members of ‘their group’ might lead to preferential learning of the caregiver’s most frequently used and/or emphasised words.

Similarly, relational functions play a role in the acquisition of first words in children. Beside learning about things and culture, they use communication as an opportunity to start an interaction and to share their inner states with others (Tomasello 1999). Existing research shows that the first words children learn include those that have the potential to initiate communication, such as calls for attention or requests (O’Grady 2005).

Prediction 2: Both children and grey parrots tend to learn words with a higher potential to elicit social interaction, such as affective expressions or words that invite others to communicate.

## Age factors influencing verbal learning

Children learn to *perceive* language from complex, non-segmented sections of speech signal, mostly not divided into words by silent pauses (see, e.g., Ramus 2002; Zacharaki and Sebastián-Gallés 2021). In contrast to perceptual learning from continuous speech their initial *production* ability is limited to isolated speech sounds (first vowel-like vocalisations several weeks after birth), syllables, and syllable reduplications, known as *babbling* (e.g., mama, baba; Molemans et al. 2012). It takes more than half a year for children to learn how to coordinate their articulation apparatus to deliver the first word-like productions, i.e., utterances that have a word-like form but no referential meaning. The first words with meaning are typically produced at 12–15 months and by 18–24 months of age children usually start to combine words (Bates and Dick 2002; Caselli et al. 2012).

According to previous research, grey parrots tend to start producing continuous speech-like utterances around 12–18 months of age, but many utter single words or phrases reportedly even before six months of age (Wright 2001). By about six months of age, parrots are therefore supposed to be able to produce speech-like structures that are longer and more complex than those observed in children. A case study by Kaufman et al. (2013) indicates that parrots can learn individual words, which they then integrate in known phrases, while other productions are learned as whole phrases. It has also been shown that they do not necessarily understand each word, as demonstrated by Cosmo’s use of long phrases containing words which were not individually present in her vocabulary and did not allow for being combined into phrases (Kaufman et al. 2013).

Parrots’ ability to produce relatively long and complex speech structures quite early on highlights a crucial difference in vocabulary acquisition between children and parrots. In children, perceptual acquisition, i.e., the ability to understand the meaning of a word (although not necessarily with exactly the same meaning as adults would) precedes the *production* of those words from a very young age (Bates et al. 1988; Bergelson and Swingley 2012). While children may understand the relevant combinatorial rules of their language quite early on (at about 8 months for word forms, and by about 18–24 months for word combinations and non-adjacent dependencies across words; Saffran et al. 1996; Culbertson et al. 2016), their own production of complex utterances is limited by the development of their vocal motor skills. Parrots, on the other hand, do not seem to need to understand the meaning of words, or the rules of combining words into phrases, to be motivated to learn and produce them (Kaufman et al. 2013). Their vocal motor constraints seem to be weaker. We can therefore formulate a third prediction:

Prediction 3: Grey parrots will produce numerically more complex multiword utterances than children.

To summarise, the predictions in our study are:

1. Children’s productive vocabularies would contain more object labels than parrots’ speech repertoires.
2. The productive vocabularies of both children and parrots would contain a relatively large proportion of words or phrases with the potential to elicit communication, for example by their emotionally expressive character or social function.
3. Parrot’s speech repertoires would include more complex multiword utterances than those of the studied children.

## Methods

### Subjects

Our sample included 21 pairs of talking parrots and children matched by vocabulary size. In the parrot sample, there were six females and fifteen males. Parrots were of various ages, ranging from 3 months to 11 years (mean age = 4.3 years, 95% CI [3.01, 5.59]). All parrots were kept as pets, acquired by their caregivers at 1–6 months of age. That means those parrots spent almost all their lives in households, alongside human family members and in some cases also alongside other pets, such as dogs or other parrots. All caregivers stated in the questionnaire that they consider their parrots part of the family. They communicate with them for 10–480 min (Mdn = 120) and cuddle with them for 10–240 min (Mdn = 60) almost every day. They also play with them for 10–240 min (Mdn = 60) almost every day, except for two caregivers who reported playing with their parrots at least once a week. Some caregivers indicated that they had deliberately taught their parrots some words, but none of the parrots had undergone any structured and systematic training. Eighteen parrots had been exposed to Czech and three to the Slovak^2^ language.

Parrots and children were matched based on their productive vocabulary size by selecting subjects from a sample of 44 children aged 8–18 months (this was a subset of the pre-pilot data in a project aimed at creating a child communication assessment tool). By ‘productive vocabulary’ we mean all words and phrases used by the children or parrots which their parents or caregivers reported as separate vocabulary entries. The final sample of children consisted of eleven girls and ten boys (mean age = 14.1 months, 95% CI [13.36, 14.84]). The children were typically developing individuals with no apparent auditory, visual, or neurological disorders, raised in a monolingual Czech language environment.

## Data collection

### Parrots

The parrots’ data were collected through an online questionnaire in 2012–2013. The questionnaire contained some questions about the parrot, other members of the household, housing and care for the bird that were not used in this study. Here, we focused on two sets of open-ended questions, the first of which began with the instruction: ‘*Please write down all human words (or phrases or sentences) which your parrot uses often, those where you believe the parrot “knows what it says”, and those that your parrot uses in an appropriate situation.*’^3^ A set of expanding items encouraged caregivers to describe, one by one, the specific situations in which each production occurred and to explain the meaning of the production (not all caregivers provided this information for all the productions). In the second set of questions, caregivers were asked to write down any remaining words or phrases which their parrots repeatedly produced, and, if applicable, indicate their specific contextual uses. Aside from that, they were asked to list all other vocal productions of their parrots, including possible environmental sounds the parrots were imitating.

### Children

The children’s data were collected in June–July 2021 during the pre-pilot phase of the Czech Communicative Development Inventory project (Jarůšková et al. 2024; Paillereau 2022). Parents were asked to make a list of all words their children understood and produced over the period of one week. The report included the gestures by which children accompanied their productions as well as stand-alone gestures. The reports were logged into an online questionnaire immediately after interaction with the child. In 36 cases (82%), the report was compiled by the mother only, in one case by the father only, in four cases by both parents, and in three cases by an unspecified caregiver.

Parents and caregivers spontaneously reported on the state of children or parrots when explaining the context or meaning of the production—that the child or parrot expressed being happy, surprised, angry, afraid, etc.

## Pair matching

The children were selected and matched to parrots based on the size of their productive vocabulary. In each case, we selected a child who represented the best pairing option with a parrot. The mean number of utterances per child was 21.62 (95% CI [14.62, 28.62]) and the mean number of utterances per parrot 20.57 (95% CI [14.06, 27.08]). In twelve cases, the difference in vocabulary size between a child and a parrot in a pair was 0–3 utterances, in five cases 4–6 utterances, in three cases 8–9 productions, and in one case 11 utterances.

## Categories

We sorted the utterances into nine categories (see Table 1 for examples). Five of those were categories for labels: labels of persons and animals, food, objects, activities and situations, and internal states. The first category consisted of names of persons and animals, including naming oneself, and of affective names. The food category included all food and drink utterances. The object category covered all objects except for foods. The activities and situations category contained all cases where the subject (infant or parrot) expressed that they did something, that the object or subject of a sentence did something, that something is non-existent, or where they referred to the state of an object or the environment. The fifth category, emotional expressions, was comprised of utterances by which the children or parrots expressed their emotional state (fear, happiness, etc.), emotions towards others, and their internal states (being hungry, thirsty, tired etc.). The sixth category, imitations of sounds, included imitated environmental sounds either by using words resembling those sounds (those that were not reported as a label) or by direct imitation of a sound. Categories 7–9 covered words used to initiate or maintain interaction with others. Specifically, category seven consisted of greetings. Category eight included utterances where the child or parrot requested that something be done (by others or by themselves). The last category, conversational expressions, covered a wider range of productions: those aimed at initiating or maintaining conversation (e.g., by asking questions), expressions of politeness (‘Please’, ‘Thank you’, etc.), as well as swearwords and utterances with unclear meaning (not explained by caregivers) possibly used to attract attention or maintain social bond.

**Table 1:** Examples of Categories

Categories	Examples of Productions
Labels of persons and animals	mama, teta (‘mom, ‘aunt’ nominative), Kubo (proper name, vocative), papoušku (‘parrot’, vocative), miláčku (‘sweetheart’, vocative)
Labels of food	oříšek (‘nut’, diminutive), vodička (‘water’, diminutive), kafíčko (‘coffee’, diminutive), ňam ňam (‘yum yum’ – explained as a label)
Labels of objects	zvonek (‘bell’), auto (‘car’), klec (‘cage’)
Labels of activities and description of situations	bác–*něco spadlo* (‘bang’ – *when something fell*), kakat (‘make a poo’), svítí (‘the light is on’), [něco] není (‘[something] is not here’), horko/horký (‘hot’), prší (‘it’s raining’)
Emotional expressions	au (‘ouch’), mám tě rád (‘I love you’), mám hlad (‘I’m hungry’), fuj (‘yuck’)
Imitations of sounds	haf (‘woof’), mňam (‘yum’—not *explained as a food label*)
Greetings	ahoj (‘hi’), dobrou noc (‘good night’), dobré ráno (‘good morning’)
Requests, commands, and induction of actions	nedělej to (‘don’t do it’), pojď sem (‘come here’), ještě (‘more or again’), ‘*I want* ‘* requests*
Conversational expressions	prosím (‘please’), jak se máš (‘how are you’), zpívá (‘sings’), swearwords, *interaction utterances with unclear meaning*

## Data analysis

We have counted the number of units that each subject produced in each category. A unit could be a single word or a multiword string. Because in the Czech language, individual words in a written text are always separated by a space, the first key we used to distinguish between a word and a multiword utterance in parent/caregiver reports was the absence/presence of a space in the reported utterance. In addition, we also considered the described meaning or context, if provided, e.g., an utterance recorded as *a a* was explained as a version of *haf haf* ‘woof woof’, so we considered it a two-word utterance. Multiword utterances were categorised as a whole as belonging to just one category. Units were annotated primarily based on the context provided by the caregivers and their common/default dictionary meaning. Multiword utterances that lacked context explanation were considered ‘interaction utterances’ (i.e., utterances used to initiate interaction) and categorised as conversational expressions. Grammatically or phonetically inaccurate productions were included, and accuracy was not coded.

A subsample of randomly selected 10 subjects (24% of the dataset) was annotated by three observers (TR, LJ, and an independent observer). Their Randolp’s kappa coefficient of reliability was 0.89 (95% CI [0.85, 0.92]). The remaining 32 subjects were annotated by two observers (TR and LJ).

**Fig. 1:**
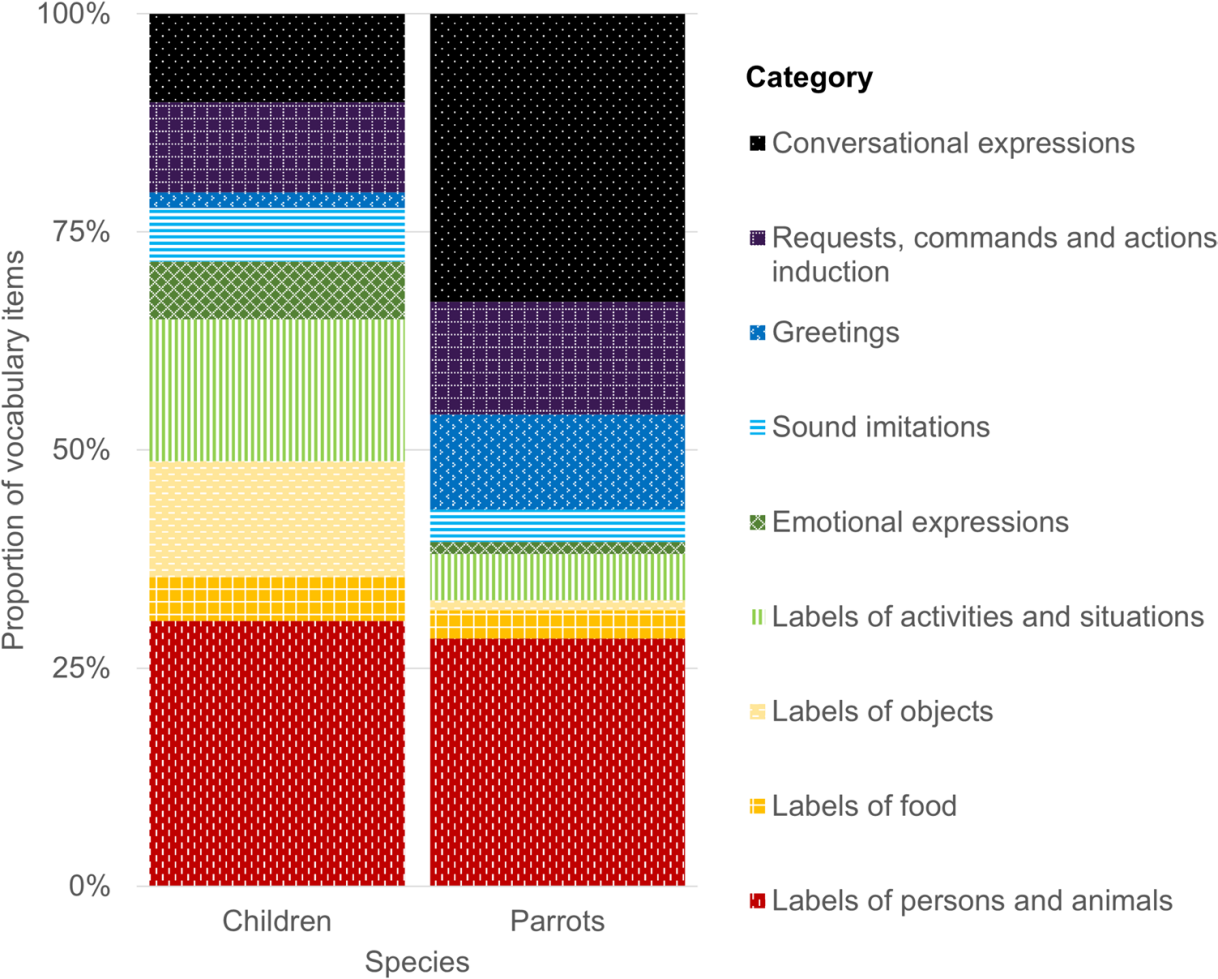
Proportional size of the nine categories per species. Categories are depicted in the same order as the legend

The counts of units in each of the nine categories (labels of persons and animals, food, objects, activities and situations, emotional expressions, imitations of sounds, greetings, requests, commands, induction of actions, and conversational expressions) were analysed using the Poisson generalised linear mixed-effects models in R (R Core Team 2022), with the *lme4* package (Bates et al. 2015). Fixed effects were the species, with a sum-to-zero contrast human vs. parrot (coded as − 1 vs. + 1), category (with eight treatment contrasts where the category Labels of persons and animals served as the reference level), and their interaction. For the fixed effect of Category, the level Labels of persons was used as the baseline because it had sufficient data for both species, its representation in the vocabulary seemed comparable for the two species (see Fig. 1) and–unlike some other categories such as Labels of objects–we did not predict any difference between the species in this area (see Introduction). The random-effects structure modelled a random intercept per subject. For our research questions, the relevant effects were the main effects of species or interactions between species and category.

A second analysis compared the count of multiword utterances across the two species. A Poisson generalised linear mixed-effects model fitted the fixed effect of species (human vs. parrot, coded as − 1 vs + 1) and a random intercept for subject. For interpretation of significance, we have adopted the commonly used alpha threshold of 0.05.

## Results

### Statistical modelling outcomes

**Fig. 2:**
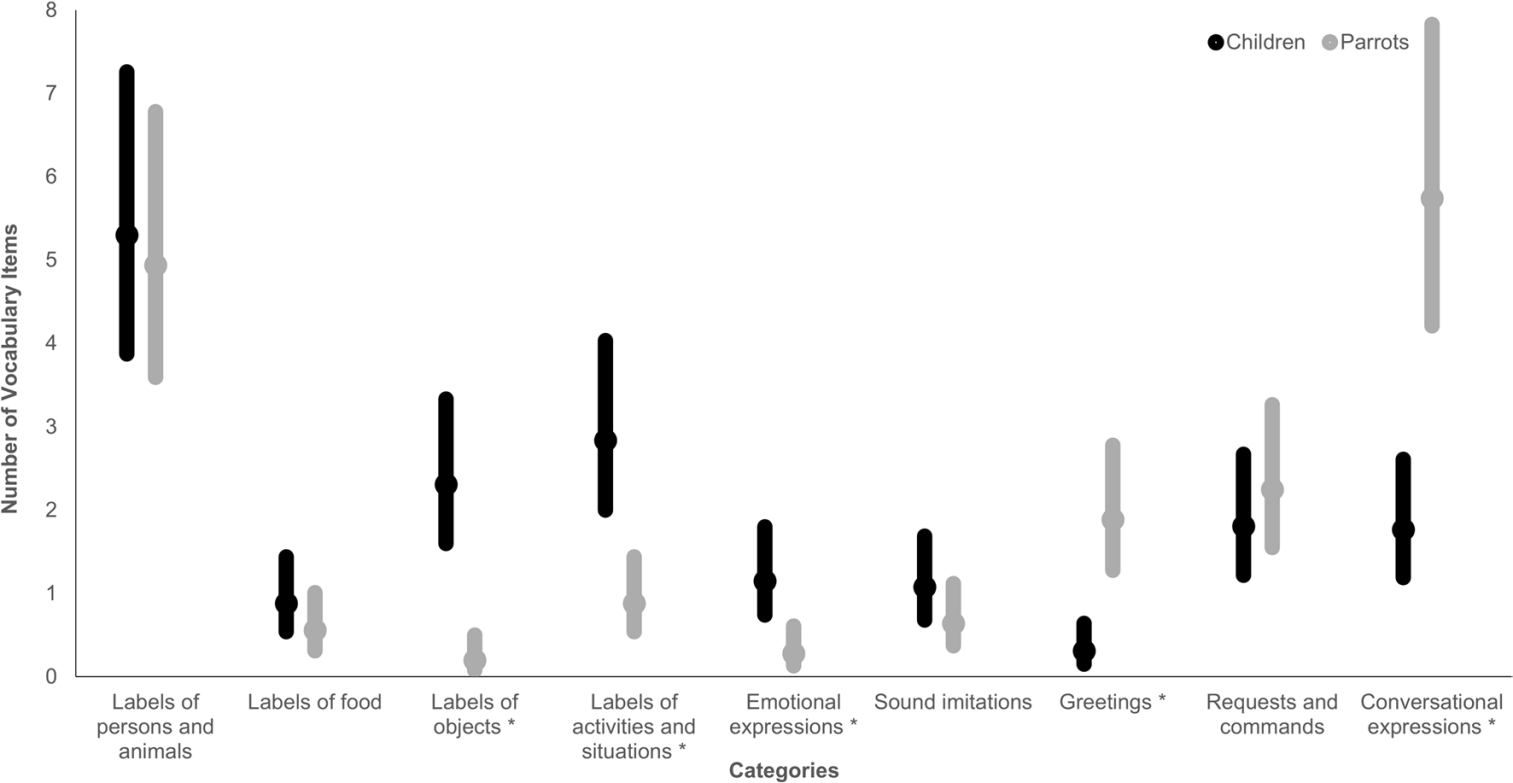
Estimated means and 95% confidence intervals of item counts for each category, in children and parrots. * indicate significant differences

Figure 1 displays the proportion of each of the nine vocabulary categories in each species; Fig. 2 shows the means and confidence intervals of the numbers of units produced by parrots and children in individual categories. With respect to the factors that are relevant for our research question, the analysis revealed significant main effect of species, and a significant interaction of species and category for five of the eight contrasts, namely those involving labels of activities and situations, labels of objects, conversational expressions, emotional expressions, and greetings (see Table 2 for fixed-effects estimates, z-values, and p-values). Pairwise comparisons (done with the *emmeans* package; Lenth et al. 2022) showed that, compared to parrots, children produced more labels of activities and situations (by about 2 units), more labels of objects (by about 2 units), and more emotional expressions (by about 1 unit), while parrots produced more conversational expressions (by about 4 units) and more greetings (by about 1 unit); see Table 3 and Fig. 2 for the estimated means and 95% confidence intervals.

**Table 2:** Fixed-effects output of the first model

Fixed effects	Estimate	SE	z	p
Intercept	1.633	0.114	14.296	< .001
Species (− children; + parrots)	− 0.036	0.114	− 0.313	.0755
Categories (referenced to Labels of persons and animals)				
Labels of food	− 1.982	0.179	− 11.081	< .001
Labels of objects	− 2.018	0.239	− 8.452	< .001
Labels of activities and situations	− 1.172	0.135	− 8.673	< .001
Emotional expressions	− 2.196	0.217	− 10.124	< .001
Sound imitations	− 1.817	0.167	− 10.879	< .001
Greetings	− 1.905	0.199	− 9.557	< .001
Requests and commands	− 0.932	0.116	− 8.053	< .001
Conversational expressions	− 0.474	0.104	− 4.552	< .001
Species X Category				
Species: Labels of food	− 0.191	0.179	− 1.069	.287
Species: Labels of objects	− 1.185	0.239	− 4.963	< .001
Species: Labels of activities and situations	− 0.549	0.135	− 4.062	< .001
Species: Emotional expressions	− 0.670	0.217	− 3.089	002
Species: Sound imitations	− 0.222	0.167	− 1.331	.183
Species: Greetings	0.943	0.199	4.730	< .001
Species: Requests and commands	0.145	0.116	1.254	.210
Species: Conversational expressions	0.625	0.104	6.000	< .001

**Table 3:** A modelled count of vocabulary units in each of the nine categories

Categories	Children		Parrots	
	Mean	95% CI	Mean	95% CI
Labels of persons and animals	5.30	[3.87, 7.26]	4.94	[3.59, 6.78]
Labels of food	0.88	[0.54, 1.44]	0.56	[0.31, 1.01]
Labels of objects	2.31	[1.60, 3.33]	0.20	[0.08, 0.50]
Labels of activities and situations	2.84	[2.00, 4.03]	0.88	[0.54, 1.44]
Emotional expressions	1.15	[0.74, 1.80]	0.28	[0.13, 0.61]
Sound imitations	1.08	[0.68, 1.69]	0.64	[0.37, 1.12]
Greetings	0.31	[0.15, 0.64]	1.89	[1.28, 2.78]
Requests and commands	1.81	[1.22, 2.67]	2.25	[1.55, 3.26]
Conversational expressions	1.77	[1.19, 2.61]	5.74	[4.21, 7.83]

The second model, which analysed multiword utterances, revealed a main effect of species (estimate = 0.355, SE = 0.154, z = 2:307, p = 0.021). It showed that, compared to children, parrots produced more multiword utterances by about 2.7 units (children: mean = 2.63, 95% CI = 1.67–4.14, parrots: mean = 5.35, 95% CI = 3.52–8.12).

## In-depth qualitative analysis of item identities

As seen in Figs. 1 and  2, labels of persons and animals formed the largest category for children and the second largest for parrots, making up 30.4% and 28.4% of their vocabularies, respectively. In parrots, a large proportion of persons’ names were names of the parrots themselves (0–4 productions, Mdn = 1). In children’s vocabularies, their own names figured only rarely (0–1 production, Mdn = 0). In three cases, children showed a tendency to overextend meanings by using *mama* with the meaning of ‘I feel bad’ or ‘food’. In one case, a child used the word *tata* ‘daddy’ to refer also to other family members.

Food labels were among the numerically smallest categories in both children and parrots, forming just 5.1% and 3.2% of the two species’ vocabularies, respectively. In our sample, children tended to use *ňam/ham*—‘yum’ (also in various forms like *mam mam, ňaňá, ňo ňo, hami*) as a universal label for food: only two of our subjects used more specific terms to refer to food. Children also tended to use a universal label for drinks. Parrots used general words for food and drinks as well (especially *vodička*, which is a diminutive of ‘water’), although three produced specific labels for their favourite treats: *piškotek, oříšek, mandlička—*diminutives of ‘biscuit’, ‘nut’, and ‘almond’, respectively. In three cases, parrots used a food label that represented a food or beverage they do not normally consume: *kafíčko* (‘coffee’, diminutive), *jogurt* (‘yoghurt’), and *maso* (‘meat’).

Object labels formed the numerically smallest category for parrots (1.4%) but amounted to 13.2% of children’s vocabulary. The most represented kinds of objects in our dataset were toys (including books), vehicles, everyday objects, and body parts. In three cases, we had reports of labelling of flowers. In the parrots’ vocabulary, objects were present only in two individuals that labelled everyday objects (e.g., *šroubovák*, ‘screwdriver’). Labels for toys were present in only one individual (e.g. *zvoneček*, ‘bell’ in diminutive). In children’s data, an utterance was in several instances interpreted by the parent as carrying multiple meanings, for instance as simultaneously a label for an object, activity with the object, and an expression of ‘wanting’ that object. Such utterances were preferentially categorised as noun labels.

Labels of activities and situations featured more prominently in the vocabularies of children (16.3%) than parrots (5.3%). Children labelled activities they executed or witnessed themselves (e.g., *haji*, ‘to lie down’ in a childish form; *oupy/hou/hoho*, ‘to swing’, *bác/ba, hop/op*, ‘bang, ups’, when they/something fell down or they tossed it) or activities that changed their environment (e.g., *tí*, imperfect form of *svítí*, ‘shines’, or *blik*, ‘blink’, when switching on lights). The most frequently reported activity labels in parrots were activities executed by someone else (e.g., *jdeme ven, pojď no pojď honem*, ‘let's go out, come on, come on quickly’ when the parrot believed the dog was going out; *mamka jede*, ‘mom’s coming’; *klepe*, 'he is knocking’) and states of environment (e.g., *prší*, ‘it’s raining’; *horko*, ‘hot’). Nevertheless, some parrots also commented on their own actions (e.g., *a jedeeeem*, ‘and here we goooo’, said when riding on a TV remote).

The category of emotional expressions formed 6.6% of children’s and 1.4% of parrots’ vocabularies. Children also reportedly expressed disfavour (seven times), pain (three times), happiness (four times), wonder (three times), affection, such as meaning ‘I love you’ (twice), and fear (once). Their expressions were mostly in the form of simple onomatopoeia (i.e., *jéje/jé/jů*,* ‘*yay’; *au*/*á* ‘ouch’). Parrots were less likely to verbally express emotions than children were. No parrot expressed happiness or affection and only one parrot had verbally expressed fear or wonder, although there were other cases in our data where parrots expressed disfavour (four times) or pain (twice). In addition to onomatopoeia (expressed by expressions such as *au*, ‘ouch’), one parrot also used a multiword utterance *my se bojíme, neboj* (‘we are afraid, don't be afraid’) to express fear. Children often expressed hunger or thirst by using standardised childish expressions such as *ham* (when eating or swallowing something), while only one parrot expressed thirst and that was done using a highly specific utterance: *a pivo a pivo* (‘and beer and beer’). Imitations of sounds were less represented in both the children (6.2%) and parrots (3.7%). In children, the category of imitations of sounds consisted mostly of words resembling the sound of something falling to the ground, of knocking, or other object and animal sounds. Two children also expressed that something was *mňam* (‘yummy’). Parrots produced various animal and object sounds.

Greetings featured about ten times more often in the parrots’ (10.9%) than in the children’s vocabulary (1.8%). At least one greeting was present in the vocabulary of every parrot in our sample regardless of the size of their vocabulary. Most used either *ahoj / ahojky* (‘hi’, 15 utterances) or *čau* (‘ciao’, 14 utterances) and one parrot even used a combination of the two, forming a novel *čahoj* (‘ciahi’). Less prominent, with one to three utterances, were *pa/papa* (‘bye-bye’), *dobrou noc* (‘good night’), *dobré ráno* (‘good morning’), *dobrý den* (‘good day’), and *nazdar* (‘hi’). Parrots also used greetings in conjunction with names (or person labels), not only their own but also the names of people and animals from the household. Among the children in our sample, only seven had a version of verbal greeting in their vocabulary. Five of these children accompanied the verbal greeting with a waving gesture. Children used fewer types of greetings than the parrots did, in most cases it was *papa* (‘bye-bye’), only three children used *aó/aji* expressions approximating *ahoj* (‘hi’). They did not use greetings in multiword utterances.

The requests and commands category was represented to a similar extent in the vocabularies of both children (10.4%) and parrots (12.9%). Children mostly used requests, mainly of the ‘I want…’ type, and all but two had at least one request in their list. This contrasts with just eight parrots who requested to be moved (and sometimes accompanied) to specific rooms and places and initiated social specific activities (playing, cuddles, kisses, bathing). Interestingly, one parrot chained words linked with water when requesting a bath, saying *koupat, prší, vodička* (‘to bathe, raining, water’). Parrots tended to utter orders and prohibitions. In 43 cases, parrots issued orders to others, including other pets, mostly dogs (e.g., *Přestaň*, ‘Stop it!’; *Pojď sem!*, ‘Come here!’; *Běž dolů!*, ‘Go down!’, or *Ticho!*,* ‘*Silence!’).

There were notable differences in the frequency of use of conversational expressions. The parrots used this category three times more often (33%) than the children (10.1%), so the difference was significant. In children, conversational expressions took mostly the form of simple ‘yes’ or ‘no’ replies and routines and games. Parrots had such a large count of conversational expressions mainly because of the higher proportion of multiword utterances with no explanation from their caregivers. We tended to categorise them as utterances whose function the parrots do not necessarily understand. This included phrases such as *Pojď sem* (‘Come here’), *Daj sem* (‘Give it here’), *Hugo čo je nového?* (‘Hugo, what's new?'), *Pepík není doma.* (‘Joey is not at home’), *Miky buď ticho*! (‘Mickey, be quiet!’). Parrots also used conversational words (such as *haló*, ‘hallo’, in phone conversation), polite expressions (e.g. *prosím*, ‘please’, *na na zdravie*, ‘bless you’), and three parrots produced swearwords. One caregiver explained that the parrot ‘can remember the swearwords best because he likes it when he repeats them afterwards and it amuses us’. They also used conversational phrases for which the caregivers believed the parrots understood how they should be used and that in the right context they would elicit response, for instance *Alenko, dáš si kafíčko?* (‘Alice, would you like some coffee?’), *Mamí, mobil!* (‘Mom, the cell phone!’), *Kam jdeš*? (‘Where are you going?’), *Co děláš?* (‘What are you doing?’), etc.

All parrots but one produced at least one multiword utterance, while 5 out of the 21 children did not produce any multiword utterances. Parrots had the longest production of six words (*Mdn* = 4), compared to five words (*Mdn* = 2) in children, and they used multiword utterances more than twice as often (in 39% of cases) than the children did (in 16.1% of cases). In children, most multiword utterances were in fact repetitions (e.g., *mňam mňam*, ‘yum yum’, *brm brm*, ‘vroom vroom’ designating car noises), while in the parrots they were mainly entire phrases or song verses. Six parrots had in their vocabulary individual words from which they formed multiword utterances or/and multiword utterances where individual words were altered. For example, one parrot used *Tak pojď, ty kozlíčku.* (‘So come on, you little goat’), *Mamku, pojď se koupat.* (‘Mommy, let's take a bath’), *Pojď se koupat, ty pusinko.* (‘Let's take a bath, sweetie’), *Tak pojď pojď, Kokoušku.* (‘So come on, come on, little Koko’), thus reusing the verb element in different utterances. Another one had both single words and changing strings of words in her vocabulary, e.g. *No poď.* (‘Come on’), *Poď sem.* (‘Come over here’), *No poď sem.* (‘Hey, come here’), *Mama, poď sem.* (‘Mama, come here’), *Mamíííí, poď sem.* (‘Mooommie, come here’), *Mama, mama, poď sem.* (‘Mama, mama, come here’), *Juro*, *Jurko* (two diminutive forms of ‘George'), *Zmizni!* (‘Get lost!'), *Juro, zmizni!* (‘Juro, get lost!’), *Poď sem, Juro!* (‘Come here, Juro!’). About 53% of the multiword utterances produced by the parrots were such that their caregivers believed the parrot used them deliberately and/or in an appropriate context.

## Discussion

In the present study, we compared the production of word-like sounds by pet grey parrots and early productive vocabularies of children aged 8–18 months. The relatively similar linguistic environment in children and parrots kept in homes as pets enabled us to compare the word categories they tend to learn and produce, and to speculate about possible communicative motivations behind the specifics of their productions. We showed that children use object labels, activity labels, and emotional expressions significantly more frequently than the parrots, while parrots use greetings and conversational expressions significantly more often than children do. Parrots also produce more multiword utterances than children with comparable vocabulary size.

Children’s frequent naming of objects might reflect their preference for toys and/or the relative abundance of toys as objects in children's environment. Toys were almost completely absent in the parrot vocabulary (except for one individual) but formed the majority of children’s object labels. In western cultural environment, toys are ever-present and often labelled by adults to children. They support exploration, which can in turn boost cognitive development (Chase 1992). Previous studies have likewise shown the importance of objects that surround children and are within their visual field, for the development of their vocabulary (Fulkerson and Waxman 2007; Waxman and Leddon 2011). The meaning, and especially the use, of objects is something that is socially learned and cooperative–and word labelling might be an important tool facilitating such social learning (Santos et al. 2002; Tomasello 1999). Parrots, in contrast, use object labels rather rarely despite their interest in playing with them (Auersperg et al 2014). It seems that focus on objects is more typical of human communication, as suggested by Tomasello (e.g., Tomasello 2011). In parrots, it can be observed in systematically trained individuals (Pepperberg 2007).

For children, focus on object labels may be a good starting point for language acquisition also for another reason: objects are clear referents, their meaning is easy to grasp, and that is why they can form a solid basis for language learning, where words with more complex meaning follow later (Gentner and Boroditsky 2001). They also enable children to share attention and communicative intention with adults, which is considered a rewarding activity for them (Tomasello 2020).

In parrots that undergo systematic speech training, such as the use of the model/rival method (see Pepperberg 2010), shared attention with a trainer might be what enables the trainer to reinforce referential learning of objects in the parrot. On the other hand, our results suggest that spontaneously learning pet parrots may not be interested in labelling objects. This might indicate that parrots perhaps do not feel the need to understand the meaning of the utterances they are learning to produce, it might reflect their lower motivation to share attention with a caregiver to objects or it might hint at their lower interest in learning about objects in general. Nevertheless, it ought to be noted that our data is based on questionnaire responses from caregivers, and we therefore cannot completely rule out the possibility that parrots do not have objects available on a regular basis or that the caregivers do not share attention to objects with their parrots.

The difference between children and parrots in labelling activities might follow a similar logic: commenting on actions and changes in the household is an opportunity to share attention with an adult and learn cultural knowledge about the environment. This seems to be pursued by children but not by the parrots. On the other hand, our results show that both children and parrots use language to follow direct goals using requests and commands. Another reason why commands feature in parrot vocabularies so much more prominently than labels of actions could be that they represent a more affective and therefore catchier version, which the parrots may be unable to distinguish semantically from mere labelling.

We can only speculate about why emotional expressions are more prevalent in children’s than in parrots’ vocabularies. One possibility is that adult humans are better at noticing and interpreting these expressions in children because their bond with own offspring makes them specifically attuned to their internal states, while it is generally difficult to detect and interpret affective states in a phylogenetically distant species, such as parrots (Anderson 2014). Parrots may also be more likely to use other, potentially innate, ‘non-verbal’ vocal signals to express their internal states. Some caregivers have reported that their parrots scream or use loud calls to express discomfort or anger, which may match the ‘squawking’ described in other groups of grey parrots, both those in captivity (Giret et al. 2012) and the wild (May 2004).

As noted above, parrots use greetings more frequently than children do. The first explanation at hand could be that greetings are the most frequent words which parrots hear, because they are used every time someone enters or leaves–and that is why they are so readily picked up. Greetings are sometimes connected with the parrots’ own names, in which case they are clearly directed at parrots by their caregivers who probably often say them in an affective tone (especially in instances such as ‘hi, honey’). That could make such words especially likely candidates for remembering and subsequent production aimed, in a reciprocal manner, at initiating communication with their caregivers, whereby the aim of such communication is to form or maintain an emotional bond with the caregiver (Colbert-White et al. 2011; Langford 2017). Still, some parrots used greetings along with names or labels of their family members (e.g., ‘granny’), including the names of pets, and they were able to imitate not only the intonation with which certain family members greet them but also their voices as such. Because the names of other family members are neither directed at the parrot nor are they the most frequent expressions they hear, it is more likely that parrots are sensitive to the affective tone of these words and their potential to initiate communication with a member of the household. In fact, some scholars link human greetings to the contact calls of other animals, including several parrot species in whose communication they play a key role (Bradbury 2003; Dahlin and Wright 2012; Todt and Naguib 2000), while parrot contact calls help individuals in a group maintain their relationships (Wanker et al. 1998). On top of that, some parrot species (e.g., parrotlets) are known to use modifications of their contact calls to individually call their relatives and particular group members. They also use their individually distinct contact calls to be identified by others (Wanker et al. 2005). In parrots, the motive of forming or strengthening an emotional or social bond could also explain their reproduction of conversational utterances such as ‘Where are you going?’ or ‘Would you like some coffee?’ In these cases, the parrots probably do not fully comprehend the meaning of their utterance, but they might understand that these utterances lead to social interaction.

In contrast to parrots, only six children used verbal greetings. Still, given that children are rarely left unattended, it may be that reunion with a caregiver need not be such a salient moment in terms of initiating communication as it is for the pet parrots, even though children may have more opportunities to use greetings because they leave the household and meet other people. Greetings can still be important to children but at a young age they may be used to expressing them with gestures, which is why they perhaps do not have much motivation to use verbal greetings. Our data shows that most children who used verbal greetings used them in combination with a gesture, which may be a precursor to the preferential use of verbal greetings (Iverson and Goldin-Meadow 2005).

As predicted, parrots used multiword utterances more often than children did. On top of that, while parrots commonly produced utterances containing various words and syllables, children’s multiword utterances often consisted of repetitions of syllables or words. Our results are in line with previous findings according to which children typically start to produce complex two-word combinations at around 18 months of age (Bates and Dick 2002), while in parrots, the age at which they utter their first verbal productions is usually around one year, although earlier productions have also been observed (Wright 2001). In fact, the youngest (three-months-old) parrot in our dataset produced utterances up to five words long. In more than half of the cases, the caregivers believed that their parrots used multiword utterances deliberately or at least in appropriate contexts. Some parrots also used both multiword phrases and the single words that made up these utterances. This may indicate that at least some of the parrots did understand that multiword utterances are composed of smaller units, and preferentially learned new phrases composed of words they already knew.

In this sense, our results are consistent with the findings of Kaufman et al. (2013), according to which parrots can learn multiword utterances both as a whole and as individual words that they combine. Still, the frequent use of conversational phrases may be related to their ability to produce complex strings of syllables without knowing the meaning of individual words or the rules that govern their combinations.

The qualitative analysis of our dataset of parrots’ productions provides additional hints indicating that pet parrots often understand at least the approximate meaning of the words they use. Some tend to preferentially use multiword utterances which are alterations of a familiar semantic and/or syntactic structure of the language they are imitating (such as in *Poď sem, Juro!* and *Mamííí, poď sem.* meaning ‘Come here’ + label of person)*.* Other parrots were inventive and produced deliberate strings of words or even new words based on known words with a similar meaning (as in *koupat*, *prší*, *vodička*, ‘to bathe, raining, water’ used to request a bath, or *čahoj*, which is a combination of two greetings *čau*, ‘ciao’, and *ahoj*, ‘hi’, thus akin to ‘ciahi’), quite possibly to underline the strength of the message.

The productive vocabulary of both children and parrots can be biased by rewards for production of certain words either in the form of food, object, interaction, or attention. It has been shown in adults that rewards increase the motivation to learn a language and even improve recall (Mason et al. 2017). Based on our data, however, we cannot say which expressions were reinforced in either parrots or children: it remains an important question for future research.

## Conclusions

Our study shows that comparably large productive vocabularies of young children and talking grey parrots differ in their composition. Compared to parrots, children use more object and activity labels, which might be a good entry point into language and cultural knowledge acquisition due to their clear referentiality to interesting and important objects such as toys. Children also use more expressions that reflect their internal state than parrots do, which may be due to the fact that humans cannot read the internal states of phylogenetically distant parrots as well as they read the states of their own children. Parrots, on the other hand, produce more conversational opening phrases and greetings than children do. This is possibly because such expressions help parrots initiate communication with their human caregivers, whereby shared communication is for parrots a way to maintain and strengthen their bond. Finally, parrots produce longer utterances, often consisting of many words. While they obviously often learn these expressions as a whole without understanding the words they are composed from, they seem to be more ready to combine words they already know than to learn completely new expressions. Moreover, some of these combinations seem to depend on understanding the meaning of the words.

The two observed groups, children and grey parrots, both live in human households where humans care for them and communicate with them. Still, their life experience inevitably differs, very possibly in ways that affect their vocabularies. Unfortunately, we have no data that would allow us to test this hypothesis and we cannot control for possible confounding factors that might lead to systematic differences in the word production of the two groups. Future research might for instance explore the role of availability of objects or naming of objects and activities during interactions with parrots by caregivers and their impact on parrot learning of object/activities labels.

On the other hand, the relative similarity of the immediate environment in which pet parrots and children live, and the high motivation and ability of parrot species to learn human vocal signals form a rare situation where it is meaningful to directly compare which categories of first words these two distant species tend to pick up from a human language. Moreover, such comparison seems to provide valuable insights into the communicative functions of first words in children and word-like sounds in parrots which, in turn, may contribute to our knowledge of the sociocognitive specifics of the human language.

To the best of our knowledge, this is the first study that systematically compares the composition of vocabularies produced by children (8–18 months old) with the productions of word-like sounds in a larger group of home-raised grey parrots. Although our results indicate a possibility of intentional word combinations in parrots, the main finding of our study is that parrots often imitate the sounds of human languages without fully engaging with some of its other key aspects, such as semantics or syntax. Our study was not, however, specifically focused on the morphosyntactic relations of parrot productions. To address this topic, future research should compare parrots’ multiword utterances with the productions of somewhat older children who have already started to combine words (e.g., children aged 2 to 3 years). Another line of future research should focus on the comprehension abilities of children and parrots. Infants’ passive vocabularies are much larger than their productive vocabularies, and children do understand the meaning of words they produce even if their semantic representation is not necessarily identical to what we find in adults. It remains to be seen whether, and to what extent, parrots generally comprehend the utterances they have in their productive vocabulary, and how their potential semantic categories compare to those of children. Cross-specific composition of perceptual vocabularies could be investigated in a perceptual experiment using the Go/NoGo paradigm or the preferential looking procedure, both of which can be adapted to the study of perception in birds and humans (e.g., Kriengwatana et al. 2016).

## Data Availability

The datasets generated during and/or analysed during the current study are available from the corresponding author upon reasonable request.

## Footnotes

1 In the Czech Republic, parental financial help is standardly provided up to three years of age of a child and mothers usually stay at home with their children until they enter kindergarten at age three. Children start daycare or kindergarten at a younger age only rarely, and there was no such case in our sample.

2 Czech and Slovak are close and mutually understandable languages (Nábělková 2007). There is therefore no reason to expect any structural differences in the vocabularies of parrots from Slovak and Czech households.

3 The original questionnaire was written in Czech language.
